# Deciphering the linguistic blueprint of DNA: context-sensitive structures, statistical patterns, and regulatory implications

**DOI:** 10.1186/s44342-025-00052-5

**Published:** 2025-10-23

**Authors:** Iskander Akhmetov, Timur Saparov, Volkan Duran, Alexander Pak

**Affiliations:** 1 Data Science laboratory, Kazakh-British Technical University, Almaty, Kazakhstan; 2 Igdir University, Igdir, Turkey

**Keywords:** DNA linguistics, Non-coding DNA, Context-sensitive grammar, Formal language theory, DNALMs, Regulatory genomics, Statistical genomics

## Abstract

DNA is often described as the “language of life” because it encodes biological information using nucleotide sequences. Unlike the traditional view focused on codon-to-amino acid mapping in coding regions, the vast non-coding genome reveals complex organizational patterns resembling natural language. This paper outlines essential approaches in DNA linguistics, including formal language theory, RNA secondary structure modeling, statistical methods, and phylogenetic analysis. Additionally, recent research on Indo-European populations shows correlations between lexical and phonemic traits and asymmetrical patterns of genetic inheritance. Together, these perspectives deepen our understanding of genome regulation, evolution, and the striking parallels between genetic and linguistic systems.

## Introduction

The comparison between DNA and language dates back to the beginnings of molecular biology. According to Francis Crick’s [1] widely recognized description, the genetic code is a form of “biological language,” with codons performing as words that transmit instructions. With the growth of bioinformatics and the incorporation of mathematical linguistics—including Zipf’s law and Shannon entropy—into genetic sequence analysis, these initiatives grew more focused in the 1990s [2–4]. Recent advances in deep learning-based sequence modeling (e.g., DNABERT, Nucleotide Transformer) and formal language frameworks now enable genome-wide investigation of regulatory patterns with linguistic structure [5–7].

Language-based understanding of DNA offers an efficient analytical framework in addition to a metaphorical one. Non-coding DNA, which comprises approximately 98% of the human genome, is still mostly unexplored, whereas coding regions of the genome have been comparatively extensively researched through classical biology. Researchers may determine the latent structures within those regions through the application of models from computational linguistics and formal language theory [4, 8]. These models can reveal statistical redundancies, long-range dependencies, and regulatory patterns.

It is important to note, however, that the linguistic analogy, despite its analytical value, is not literal. Unlike natural language, DNA has no communicative intent, semantic layers, or cognitive context. The meaning of genomic elements is shaped by biochemical interactions, not syntactic structure. In this review, these limitations are acknowledged and the linguistic approach is treated as a heuristic tool rather than a direct biological equivalence.

The field has been further revitalized by newest developments in artificial intelligence, particularly in the area of natural language processing. Language models like BERT and GPT, which initially were established for human text, are now being modified for genomic data [5], providing new insights into the regulation syntax of DNA. Through modified gene expression prediction, functional motif authentication, and comprehension of progressive mechanisms, these interdisciplinary approaches pave the way for new advances in genomics [5, 9].

Since DNA uses a sequential arrangement of nucleotides to encode the instructions for life, it has long been compared to a language. The genetic code is traditionally understood as a fixed lexicon in which amino acids are represented by codons. However, only a small portion of code regions are covered by this viewpoint. The structural complexity of non-coding DNA, which makes up almost 98% of the human genome, reflects the context-sensitive and hierarchical character of human language [10]. To uncover the genome’s underlying grammar, recent multidisciplinary research has used formal language theory in conjunction with statistical analysis. Additionally, genetic-linguistic studies—like those looking at Indo-European populations [11]—indicate that language and gene evolution might be more connected than previously believed.

Modern transformer-based architectures such as DNABERT and GROVER have been adapted to genomic data and are trained to capture statistical and structural regularities, including Zipf-like distributions and long-range dependencies [12]. This review aims to integrate these diverse insights into a cohesive framework, advancing our understanding of DNA linguistics.

## Formal grammars and RNA/DNA structures

The symbolic nature of genetic sequences makes them a natural object for analysis using formal languages. Modeling DNA and RNA as linguistic objects allows us to identify both regular and nested and context-dependent patterns underlying their structural and functional organization. The linguistic approach to biological data uses different classes of grammars, which differ in their level of expressiveness. Context-free grammars (CFGs) are used to describe nested structures, such as stem-loop formations in RNA, which are hierarchically organized but obey fixed rules. Context-sensitive grammars (CSGs) have greater expressive power, allowing for positional and structural variability, such as splicing sites or regulatory motifs. Tree-attached grammars (TAGs), which evolved from the theory of natural language syntax, allow cross-dependencies and pseudo-nodes to be formalized while remaining algorithmically controllable.

Along with grammatical formalisms, statistical methods are actively used in genomic linguistics. Zipf’s law, found both in natural language and in non-coding regions of DNA, describes the distribution of element frequencies by rank, following a power law, indicating the presence of a hidden hierarchy. Shannon’s entropy, which measures information redundancy, allows the degree of structured organization to be quantified: as a rule, lower entropy in non-coding regions indicates potential functionality.

This theoretical basis is used for sequence modeling in the following sections. Below, we consider the key formalisms used in bioinformatics to describe and analyze the structural features of DNA and RNA.

### Formal language theory and context sensitivity

Formal language theory provides a rigorous mathematical framework for describing symbolic systems such as DNA and RNA sequences. Since the introduction of string operations to study biological recombination processes (e.g., head’s splicing system [13]), formal grammars have played a crucial role in molecular bioinformatics. Zolyan [10], in particular, proposed interpreting the genetic code as a context-dependent grammar, where the functional role of a nucleotide determined its position in a codon and a local context. One of the first to propose linguistic modeling of biological data was Searls [14], who developed the framework of “DNA linguistics”. He showed that biological elements—motifs, linkage sites, and regulatory mechanisms—can be viewed as syntactic categories, and their interactions as production rules. This approach relies on formal grammars, ranging from regular to context-sensitive, and allows modeling the symbolic and hierarchical organization of DNA sequences.

In current research, various types of grammars are used to represent different levels of biological structure. Regular grammars suffice for modeling short tandem repeats, while CFGs are used to describe nested RNA structures. CSGs are required when the meaning or role of an element depends on its surrounding context, as in splicing recognition. More advanced formalisms like TAGs support the modeling of long-distance and cross-serial dependencies, including pseudoknots.

For example, regular grammars may be sufficient to describe repetitive elements or tandem repeats, while nested structures such as stem-loops in RNA require context-free grammars. More sophisticated mechanisms, such as stochastic grammars and tree-joining grammars, have been used to capture long-range dependencies and pseudo-nodes. Recent advances in biological data grammars (e.g., [6]) can rapidly and automatically learn these formal representations from annotated genomic corpora.

However, formal grammars face limitations when applied to biological phenomena: DNA semantics, unlike language, is shaped by biochemical interactions rather than communicative intent. Furthermore, non-linear interactions (e.g., chromatin loops, epigenetic modifications) often elude purely syntactic modeling. Therefore, grammar-based approaches are increasingly combined with statistical and neural models for balance structure and generalization.

While the analogy between DNA and natural language is valuable for revealing structural principles such as hierarchy, context sensitivity, and redundancy, it is equally important to recognize differences that limit this metaphor. Unlike human languages, DNA does not encode communicative intent, semantic nuance, or adaptive feedback. Its “meaning” arises from biochemical interactions and evolutionary constraints rather than symbolic reference. In language, syntax evolves through social interaction, whereas in the genome, it is determined by molecular constraints and the history of mutations. These differences imply that linguistic formalisms, while useful for describing organizational logic, cannot fully explain functional dynamics without incorporating biochemical and evolutionary models.

This also highlights the conceptual limits of linguistic analogies: although they are useful for describing the structure and logic of sequence organization, they are unable to fully capture the biological specificity conditioned by molecular and evolutionary mechanisms. Such approaches must therefore be complemented by empirical biological interpretations.

According to these linguistic models, elements like splice sites, enhancers, and promoters may function in accordance with implicit syntactic rules, revealing structural similarities between natural languages and genomic organization. By using these grammatical formalisms, it is easier to examine the organizational logic found in non-coding DNA, which was formerly written off as “junk” but is now increasingly seen as functionally essential.

In addition, frameworks like Dyck languages and locally testable languages offer a theoretical foundation for modeling evolutionary shifts from straightforward linear sequences to intricate, hierarchically structured genomic architectures. By highlighting DNA’s generative, rule-governed characteristics, this viewpoint supports the idea that it is a biologically encoded language.

To mimic the contextual diversity seen in spoken language, an adenine in the first position can have a different regulatory function than an adenine in the second place. Primitive DNA may have developed from simple linguistic systems before becoming more complex, according to theoretical structures like locally testable and Dyck languages [15].

#### Example 1

(Modeling splice sites using context-sensitive grammar) The example of eukaryotic **splice sites**, which are essential for RNA splicing, helps to illustrate how formal grammars relate to genomic sequences. The following pattern is frequently seen at a canonical donor site: AGGT...CAG where GT marks the start of the intron and AG the end. In a *context-sensitive grammar (CSG)*, we can define production rules that consider surrounding nucleotides (the “context”) to assign functional roles:

- Terminal symbols: $$\Sigma = \{\texttt {A}, \texttt {C}, \texttt {G}, \texttt {T}\}$$
- Non-terminal categories: Exon, Intron, Donor, Acceptor, etc.

A simplified CSG might include rules like:

Exon AG Donor -> Exon’ AGGT

Donor... CAG Acceptor -> Intron CAG Exon

These rules indicate that a donor site (AGGT) only emerges if preceded by an exon and followed by an intron. The recognition of the donor depends on its position and neighboring symbols—a property that context-free grammars cannot capture. Thus, **CSGs allow us to model how biological “meaning” arises from specific sequence environments**, mimicking syntactic context in human languages.

In recent models like GENA-LM [16], regulatory patterns are encoded with both sequential embeddings and implicit grammatical cues derived from functional annotations, revealing a hybrid space between data-driven and rule-based representations.

This modeling logic can also be extended to other regulatory elements, such as promoters or enhancers, where motif function depends on surrounding composition and location.

### RNA structure modeling and tree-adjoining grammars

RNA molecules pose a challenge to conventional context-free language models because of their intricate secondary structures, such pseudoknots. These non-context-free dependencies have been successfully captured by tree-adjoining grammars (TAGs), which offer a formal basis for expressing the overlapping and nested structures found in RNA [15]. TAGs, which were first created in computational linguistics to simulate cross-serial dependencies [17], demonstrate the close ties between linguistic theory and molecular biology by providing useful tools for RNA structure prediction in addition to showcasing the generative potential of linguistic frameworks.

The ability of tree-adjoining grammars (TAGs) to simulate cross-serial dependencies and nested long-distance interactions—two essential features of RNA pseudoknots and structural motifs—is one of their primary benefits. TAGs are especially helpful in the parsing and annotation of complex RNA topologies because they provide more expressive power while keeping computational tractability when compared to conventional context-free grammars (CFGs).

To predict RNA secondary structures and examine conserved motifs in structured RNAs like tRNAs, ribosomal RNAs, and regulatory non-coding RNAs, TAG-based models have been used in real-world applications [18]. To make predicted structures more biologically plausible, some bioinformatics tools combine thermodynamic or evolutionary constraints with grammar-based parsing.

Moreover, new methods imply that combining deep learning architectures with symbolic grammars like TAGs could produce hybrid models that can achieve both statistical generalization and structural accuracy [6]. As part of a larger effort to unravel the formal rules—syntactic and functional—that dictate the behavior of nucleic acids, these advancements suggest a unified framework for comprehending RNA folding and function.

### DNA as hypertext and motif-based encoding

The genome is more than just a straight string of nucleotides; it is organized like a hypertext [19], with regulatory elements (insulators, promoters, and enhancers) interacting over vast genomic distances. These non-coding sequences modify gene expression in a context-dependent way, acting as connectors or punctuation. Modern motif-oriented models, like MoDNA [20], improve the identification of functional binding sites and the extraction of regulatory signals by incorporating well-known regulatory patterns into the pre-training phase. The increasing complexity of DNA language models is highlighted by benchmarking frameworks like as DART-Eval [9], which assess these models on tasks like motif finding and variation effect prediction.

In the same way that hyperlinks in digital text link semantically related but spatially separated elements, genomic elements such as enhancers, silencers, and insulators interact with target genes over great linear distances to form a dynamic three-dimensional regulatory network. This analogy goes beyond hypertext’s structure to include its function. Similar to a syntactic system, these connections are frequently mediated by particular motifs that function combinatorially, meaning that a motif’s meaning and impact are determined by its context, order, and co-occurrence with other regulatory elements.

Recent developments in motif-based pretraining (e.g., MoDNA) show that adding known regulatory syntax to model architectures greatly increases predictive accuracy for tasks like transcription factor binding site localization and enhancer-promoter interaction prediction. This change reflects a more general conceptual shift away from viewing DNA as a flat sequence of symbols and toward viewing it as a semiotic system, in which patterns gain regulatory “meaning” based on their structure, position, and history of interactions. Accordingly, the genome can be viewed as both narrative and code: a modular, recursively structured composition in which textual elements known as regulatory motifs form a cohesive, though non-linear, message.

## Statistical differences between coding and non-coding DNA

Compelling evidence suggests that non-coding and coding regions of DNA sequences exhibit distinct linguistic features, based on the statistical properties of these sequences. Linguistic analysis of DNA shows that non-coding sequences exhibit long-range correlations akin to those in natural language, while coding sequences are generally less correlated. When applying Zipf’s law and entropy-based analyses to genomic data, this distinction becomes especially clear.

Zipf’s law, originally formulated for word frequencies in human language, has also been applied to genomic sequences by treating nucleotide n-grams as words. Early studies observed Zipfian distributions in non-coding DNA regions [4, 21], suggesting that these regions may carry structured regulatory information rather than being merely “junk DNA”. Research suggests that non-coding DNA exhibits a power-law distribution in n-gram frequencies, similar to the word distributions found in human texts. The redundancy in non-coding regions is significantly greater than that found in coding sequences, indicating that non-coding DNA may serve as a structured regulatory language rather than simply being “junk DNA.” Calculations of Shannon entropy [2] provide additional backing for this hypothesis, indicating that non-coding DNA has lower entropy and greater redundancy than coding DNA. Recent models evaluate sequence compressibility and entropy with attention-based attribution scores and cross-entropy loss [7], further supporting the interpretation of low-entropy non-coding regions as a form of structured, functional code. This is also consistent with hybrid transformer models such as GENA-LM [16], which implicitly encode such structure via long-range dependencies.

## Comparative overview of analytical models

To capture the linguistic characteristics of genomic sequences, a variety of analytical techniques have been developed. These methods range from sophisticated deep learning-based architectures to traditional statistical tools. Although the theoretical presumptions, interpretability, and computational requirements of each technique vary, they all provide distinct insights into the structure and function of DNA. Selecting the best approach for particular biological tasks requires an understanding of these distinctions.

Table 1 offers a comparative overview of the various analytical techniques used in DNA linguistics. It outlines the kinds of data that these approaches deal with, the underlying theories, and the benefits and drawbacks of each in real-world scenarios.

**Table 1:** Comparison of analytical methods in DNA linguistics

Method	Data type	Principle used	Strengths	Limitations
Zipf Analysis	Coding/non-coding	Rank-frequency distribution	Reveals hierarchical structure	Sensitive to window size
Entropy Calculation	DNA sequences	Information theory	Measures redundancy and complexity	Requires long sequences
Tree- Attachment Grammars (TAGs)	RNA structures	Formal grammar	Captures nested dependencies	Complex to implement
DNABERT	Whole genome	Deep contextual embeddings	Learns long-range dependencies	Requires large datasets and GPUs
MoDNA	Regulatory regions	Motif-based transformer	Improves biological relevance of embeddings	Limited to known motif sets
Hybrid Symbolic-Neural Models	Regulatory regions	Integration of formal grammars with neural models	Combines structure with statistical generalization	Requires complex training and interpretation

Adapted from Patel et al. [9]; Ji et al. [5]; An et al. [20]

In addition to the analysis of individual sequences, the presented methods allow for consistent cross-domain and cross-species studies. They can be applied to different genomic taxa as well as to natural language corpora, using the same metrics (e.g., Zipf’s law indices, entropy profiles, motif redundancy coefficients). This approach ensures data comparability between biological and linguistic systems. The integration of formal grammatical models such as TAG allows for the identification and quantification of nested dependencies in DNA and human languages. Examples of the application of these comparative methods to different species are given in Section 9.

## Zipf’s law and structural efficiency in DNA

Linguistic laws, originally formulated in the study of human language, have been found to apply to biological systems, particularly at the molecular level. Several key linguistic laws are relevant in the context of DNA analysis:

### Zipf’s rank-frequency law

According to Zipf’s rank-frequency law, an element’s frequency is inversely related to its rank. This law has been noted in oligonucleotide distributions, gene expression levels, and protein superfamilies within genomic sequences. While early studies supported the presence of Zipfian patterns in genomic data [4], more recent work [22] suggests that Zipf’s law may vary across genomic regions and species, emphasizing the need for careful scale selection. The emergence of Zipfian distributions in DNA supports the hypothesis that genetic information may be organized hierarchically, resembling the structure of human language.

When using Zipf analysis on n-gram distributions in non-coding DNA, this phenomenon is particularly noticeable. Researchers have found that when fixed-length nucleotide sequences (like 3-mers or 4-mers) are treated as linguistic units, their frequencies exhibit a power-law trend that is comparable to that of human languages. These distributions point to a combinatorial structure at the heart of genomic regulation, where motifs that appear frequently may be crucial for chromatin accessibility or transcriptional control. Additionally, studies conducted across species have verified that Zipfian behavior persists in non-coding regions, suggesting a potential universal mechanism of hierarchical redundancy-based biological information encoding [8].

### Zipf’s law of abbreviation

Another relevant linguistic law is Zipf’s law of abbreviation, which posits that more frequently used words tend to be shorter. In DNA, frequently occurring regulatory motifs and transcription factor binding sites tend to be shorter than less common motifs, mirroring the principles observed in natural language.

According to empirical research, conserved regulatory motifs like CpG islands and TATA boxes frequently show both high frequency and structural compactness [23]. Their briefness may lower the metabolic cost of transcriptional regulation and improve binding specificity. Moreover, this tendency to abbreviate is not exclusive to individual motifs; whole classes of small RNAs, like microRNAs, exhibit a similar pattern and operate effectively because of their small size and high genome-wide recurrence.

The idea that biological systems, such as human language, are subject to evolutionary pressures favored informational efficiency—condensing high-frequency signals into shorter, more manageable units without compromising regulatory complexity—is thus supported by the genomic implementation of the abbreviation principle. Additional linguistic laws such as Heaps’ law, which connects vocabulary growth to sequence length, have also been adapted to DNA [24], highlighting constraints on motif reuse.

### Menzerath’s law

Menzerath’s law refers to the inverse relationship between the size of a whole and that of its parts. In the field of genetics, this law is exemplified by the tendency for genes that have a greater number of exons to possess shorter individual exons. Analogously, in RNA structure, it is observed that larger RNA molecules generally possess smaller constituent domains, suggesting an optimization principle that corresponds with linguistic efficiency.

Non-coding genomic regions have also been shown to exhibit this inverse scaling behavior. For instance, smaller individual hairpin or stem-loop motifs typically make up long non-coding RNAs (lncRNAs) with complex secondary structures [25]. Likewise, transcripts with more isoforms tend to have shorter average exon lengths in alternative splicing events, indicating that structural simplicity at the component level may be sacrificed for regulatory versatility. These trends support Menzerath’s law and suggest that biological systems, such as human language, use smaller, modular components sparingly to achieve complexity. This principle appears to be a general constraint influencing the evolution of informational and regulatory systems because it is repeated across various molecular layers, including genes, RNAs, and protein domains.

These linguistic laws underscore the deep structural parallels between genetic information processing and human language, further reinforcing the hypothesis that non-coding DNA functions as a complex regulatory code rather than non-functional genetic material.

## Power-law behavior and redundancy patterns

### Long-range correlations and the DNA walk

Statistical analyses have revealed that non-coding DNA exhibits scale-invariant properties reminiscent of natural language. The DNA walk method maps nucleotide sequences into one-dimensional trajectories based on the alternation of purines and pyrimidines [3, 8], uncovering long-range correlations in non-coding regions with scaling exponents ($\alpha \approx 0.6$–0.9). In contrast, coding regions typically show $\alpha \approx 0.5$, indicating uncorrelated behavior. These long-range dependencies suggest that non-coding DNA maintains a regulatory “memory” over kilobases, analogous to long-range syntactic dependencies in language.

These results support the notion that the genome, especially its non-coding regions, is not a random collection of nucleotides but rather displays self-similar patterns across scales and a fractal-like organization. Similar power-law dependencies are frequently found in linguistic corpora, where sentence structures and thematic progressions exhibit the scale-invariant behavior uncovered by DNA walk analyses. By permitting enhancer-promoter interactions or preserving chromatin architecture, for example, these long-range correlations may enable coordinated regulation across distant genomic loci in a biological sense. This supports the idea that non-coding DNA forms a multilayered regulatory language similar to discourse-level structures in natural language by encoding both global organizational signals and local motifs.

The hypothesis of similarity between non-coding DNA and natural languages was tested using a comparative analysis of key statistical parameters of both systems. The volume of DNA sequences used provided sufficient statistical power to detect long-term correlations and structural patterns in different types of sequences. To compare key statistical characteristics, three main metrics were chosen: the Zipf exponent, the Hurst coefficient, and the Shannon entropy. Data from various studies reflecting the properties of the English language were used as a language corpus.

Table 2 shows that the Hurst coefficients for non-coding DNA and the English corpus are in very close ranges, indicating the presence of similar long-term correlations and scale-invariant structures in both systems. This is consistent with the idea of a complex, hierarchical organization in both genomic sequences and natural languages.

**Table 2:** Comparison of statistical indicators of non-coding DNA and the English language

Metrics	Non-coding DNA	English (corpus)	Source
Zipf exponent ($\zeta$)	0.2385	$\sim$ 1.0	[26]
Hurst coefficient (*H*)	0.6811	$\sim$ 0.67–0.74	[27]
Shannon entropy (bits/symbol)	1.99	$\sim$ 1.58	[28]

At the same time, the Zipf exponent, reflecting the power law of trigram frequency distribution, is significantly lower for DNA, which is associated with a more limited combinatorial complexity due to the smaller alphabet size and biological limitations. The Shannon entropy value is higher for non-coding DNA, which is associated with the peculiarities of nucleotide distribution and the limited number of symbols in the alphabet (four nucleotides versus tens of thousands of words in a language).

Thus, despite the coincidence of some mathematical patterns, the functional nature and biological role of non-coding DNA and natural language differ significantly. This quantitative cross-domain comparison allows us to go beyond qualitative analogies and create more substantiated hypotheses about the “linguistic” nature of genomic sequences.

### Zipfian distributions and n-gram entropy

Analysis of k-mer frequency distributions in non-coding DNA regions shows that they often obey a Zipfian law, i.e., exhibit a power-law decay in n-gram frequency. This frequency structure, similar to distributions in natural languages, indicates high redundancy and structural variability in non-coding regions. This is consistent with the hypothesis of the presence of a latent regulatory grammar in non-coding DNA that can balance variability and functional stability. Modern transformer models such as DNABERT and GROVER exploit these statistical properties to learn context-sensitive representations. In particular, the GROVER model applies byte-pair encoding (BPE), which relies on Zipf-like frequency distributions to construct a balanced dictionary of k-mers [12]. This highlights the practical importance of Zipf-like patterns for representing sequences in bioinformatics models.

Furthermore, non-coding regions typically exhibit lower Shannon entropy compared to coding regions, indicating the presence of internal structure, repetitive functional motifs, and significant redundancy. Despite the apparent low information density, such an entropy pattern may reflect a highly constrained regulatory code optimized for mutation robustness and high learnability in neural network models. These repetitive elements are likely the result of evolutionary selection to ensure robustness of regulatory information transmission. In contrast, coding regions optimized for accurate and diverse translation are characterized by higher entropy and less pronounced Zipf-like patterns. Thus, non-coding DNA can be viewed as a semiotic system with grammatical regularities capable of encoding regulatory logic beyond the linear order of nucleotides. These differences are confirmed by the power-law decay of n-gram frequencies and lower entropy. A general comparison of these characteristics is presented in Table 3, which compares the linguistic properties of coding and non-coding DNA regions.

**Table 3:** Comparison of linguistic features in coding vs. non-coding DNA

Feature	Coding DNA	Noncoding DNA
Correlation exponent ($\alpha$)	$\sim$0.5 (uncorrelated)	$\sim$ 0.6–0.9 (correlated)
Zipf exponent ($\zeta$)	Logarithmic decay	Power-law decay
Entropy	Higher	Lower
Redundancy	Lower	Higher

Table 3 compares coding and non-coding DNA using four linguistic parameters: correlation index, Zipf distribution pattern, entropy level, and redundancy level. These metrics highlight that non-coding DNA can carry structured regulatory information beyond its protein-synthesizing function. Models borrowed from the field of natural language processing (NLP) are most often used to identify and exploit such hidden patterns.

To confirm the statistical reliability of such patterns, well-established quantitative methods are used in the literature. The parameters of the power distribution are estimated using the maximum likelihood method with the Clauset, Shalezi, and Newman correction [29], which allows avoiding systematic distortions typical of the logarithmic approximation. To check the adequacy of the Zipf model, the Kolmogorov–Smirnov statistics are used, as well as the AIC and BIC information criteria, which allow comparing Zipf-like distributions with lognormal and exponential alternatives [30, 31]. To assess the stability of the parameters, bootstrapping with up to 10,000 iterations is often used, which is especially important when analyzing short or fragmentary sequences. Additionally, a comparison is made with null models—random permutations and first- and second-order Markov chains—to exclude the possibility that the identified patterns are trivial consequences of local dependencies.

These procedures significantly improve the reproducibility and statistical validity of inferences about the presence of linguistic structures in non-coding regions of DNA.

### Limitations of computability and scalability of grammars

Applying formal grammars to genomic data requires taking into account their computational complexity and scalability. Regular grammars create a linear time structure and are effective for detecting simple repetitive structures. Context-free grammars (CFGs) require polynomial time ($O(n^3)$), while tree-adjoining grammars (TAGs) and context-sensitive grammars (CSGs) can have complexity of $O(n^6)$ and higher, which makes them difficult to use when analyzing long stretches of DNA.

To circumvent these limitations, approximate methods are used: windowing, heuristics, sequence fragmentation, as well as hybrid constructions combining grammar rules and neural network components. This approach allows maintaining a balance between accuracy and scalability, which is especially important when analyzing non-coding regions.

## NLP-based models and their applications

The underlying regulatory syntax of the genome is captured by DNA language models (DNALMs), which are the result of recent deep learning techniques. For example, DNABERT overcomes the drawbacks of conventional CNN and RNN models by leveraging Transformer architectures to capture long-range nucleotide dependencies [5]. Beyond DNABERT, models like DNABERT-2 [32] and GENA-LM [16] implement subword tokenization and memory-augmented architectures to model longer sequences with regulatory precision. The Nucleotide Transformer [7] scales to hundreds of species, revealing conserved features through multilingual embedding spaces. Furthermore, biological priors are incorporated into motif-oriented frameworks like MoDNA to enhance learnt representations, especially in regulatory regions. To thoroughly assess these models on tasks like variation effect prediction, motif detection, and promoter prediction, benchmarks such as DART-Eval have been developed [9].

In addition to benchmark accuracy metrics, an important indicator of DNALM performance is their ability to generate biologically valid predictions. Recent studies have demonstrated that predictions made by DNA language models, such as DNABERT and MoDNA, are experimentally validated. For instance, Kim et al. [33] proposed the CWAS-Plus framework, which integrates a DNABERT-based model to prioritize non-coding regulatory mutations associated with complex diseases. The predicted risk variants and regulatory regions were validated both in vitro and through functional genomics assays. A follow-up study further applied this methodology to multiple cell types, identifying functionally relevant enhancer regions and non-specific variants [34]. Similarly, MoDNA showed strong performance in locating transcription factor binding sites, confirmed using ChIP-seq data. These examples illustrate how DNALMs, grounded in NLP methodologies, can prioritize disease-associated mutations and uncover functional non-coding elements, underscoring their biological relevance and practical utility.

Although DNALMs capture extensive contextual information, recent comparative assessments suggest that in some cases, simpler ab initio models may outperform them, highlighting the need for further refinement and biological integration.

### Applications of DNA linguistics

The practical applications of DNA linguistics are extensive and span several fields of biotechnology and biomedical research. By discovering disease-associated regulatory concepts that conventional techniques might fail to identify, researchers can improve diagnostic precision by comprehending the structural, grammatical, and statistical characteristics of non-coding DNA. By allowing for a more precise prediction of variant effects based on learned contextual patterns, language-based models also aid in the development of genetic tests.

DNA linguistics assists in target selection in genome editing, especially in CRISPR-based systems, by locating areas that use distinct motif syntax. This lowers off-target effects and increases efficiency. Additionally, by identifying subtle, language-like differences in non-coding regions that reflect progressive divergence, linguistic analysis contributes in phylogenetic studies and species classification. These applications demonstrate how linguistic frameworks support practical developments in evolutionary genomics, synthetic biology, and personalized medicine in addition to enhancing theoretical knowledge of the genome.

Recent studies further indicate that language capability transfer from natural language models to genomic sequences may enhance model performance, opening new avenues for research. Beyond structural modeling, regulatory logic influenced by evolutionary constraints may be reflected in linguistic patterns found in noncoding DNA. For example, low-entropy motifs and Zipfian distributions frequently match transcription factor binding sites, suggesting possible roles in the regulation of gene expression. These trends imply that the robustness, redundancy, and modularity of biological regulation may be influenced by linguistic characteristics.

Practical applications of this method include better synthetic promoter design, improved CRISPR target selection, and more precise prediction of disease-associated variants. Precision medicine and logical genome engineering can both benefit from the ability of NLP models trained on genomic sequences to identify minute regulatory patterns.

In recent years, the application of linguistic models has expanded to previously poorly understood mutation phenomena such as somatic non-coding variations, particularly in a variety of neuropsychiatric disorders. Ahn and Kim [35] discovered the contribution of brain somatic mosaicism to schizophrenia and used basic linguistic models to reveal the workings of tissue-specific mutation patterns. These approaches exploit the potential of linguistic DNA analysis not only to study heritable variations but also to diagnose and treat diseases caused by somatic mutations. This is an extension of the scope of DNA linguistics to include the study of complex and tissue-specific regulatory principles.

### Visualization and interpretation of genomic grammars

To bridge the gap between abstract representations and biological insight, visualization tools play a crucial role in interpreting genomic grammars. Attention heatmaps from Transformer-based models can highlight regulatory motifs or long-range dependencies. Grammar-based parsers may visualize syntactic structures in non-coding regions, similar to parse trees in natural language. These tools enhance explainability and foster collaboration between computational scientists and molecular biologists.

Aside from attention maps, other visualization formats that show distinct aspects of genomic structure are k-mer Zipf plots, motif density heatmaps, and entropy landscapes. Entropy landscapes, for instance, can show areas of limited variability that might be associated with functionally significant loci, and motif co-occurrence graphs can reveal the combinatorial logic between transcription factor binding sites. In addition to being easier to understand, these representations help produce biologically significant hypotheses, like the existence of insulator boundaries, splicing regulatory elements, or cryptic enhancers. Interpretability will be a key factor in the development of future DNA language models since visual analytics will be crucial in converting latent representations into testable biological insights as models become more complex.

### Synthetic genomics and artificial code design

Besides theoretical understanding, DNA linguistics has the potential to lead to useful advancements in synthetic biology. The logical design of artificial promoters and gene circuits is made possible by an understanding of the grammatical structure of DNA [36]. Using motif libraries and context-aware rules, synthetic DNA constructs can be designed to maximize regulatory behavior, much like language, which is governed by syntactic and semantic rules. By lowering off-target effects and enabling programmable control of gene expression, this grammar-based method improves the predictability, modularity, and safety of gene editing systems. The creation of artificial genomes and intelligent biocomputing platforms are among the new bioengineering possibilities made possible by the incorporation of linguistic constraints into DNA synthesis.

## Co-evolution of genes and languages

Complex correlations between genetic markers and language traits have been demonstrated via empirical research. Studies of Indo-European populations show that maternal lineages (mtDNA) are more strongly associated with phonemic traits [11], while paternal lineages (Y-chromosome markers) are more strongly associated with lexical traits. A growing number of studies now attempt to align linguistic phylogenies with population genomics, using co-inference techniques that leverage shared methods such as tree reconciliation and motif alignment [37]. The idea that genetic inheritance and language evolution are linked is supported by this asymmetric relationship, which suggests that sex-specific migration and social dynamics have an impact on language evolution. These associations offer strong support for a co-evolutionary process, according to which our genetic past plays a role in the mechanisms underlying language transmission.

Genome-wide research has further supported this theory by showing that, especially in areas with limited admixture or long-term population stability, linguistic boundaries frequently correspond with genetic discontinuities. Language transmission may act as a barrier to gene flow, as evidenced by the strong correlations found between genetic clusters and linguistic isolates in the Caucasus and parts of the Himalayas. On the other hand, lexical characteristics frequently endure despite changes in genetic composition in regions impacted by significant conquests or migrations, such as the Indo-European expansion, indicating that language can also spread through cultural diffusion apart from complete demographic replacement.

Furthermore, the interaction between language and genetics is mediated by cultural transmission. Linguistic and genetic patterns can be separated by historical phenomena like language shift, elite dominance, and bilingualism. For instance, while genetically homogeneous groups may show notable linguistic divergence, some language isolates endure in genetically mixed populations. These findings highlight the fact that social, political, and environmental factors, in addition to biological inheritance, have an impact on language evolution.

## Cross-species comparison of genomic grammars

Comparing genomic “languages” across species is one productive avenue for future study. Similar hierarchical and context-sensitive patterns have been found in other eukaryotes, including yeast and mice, despite the complex regulatory grammars found in non-coding regions of human DNA. On the other hand, prokaryotic genomes, such as those of bacteria, frequently have less regulatory “redundancy” and simpler, more straightforward coding structures. This discrepancy points to a potential evolutionary gradient of linguistic complexity, in which organismal complexity causes regulatory grammar to grow. It may be possible to discover universal principles of genomic organization and evolution by examining conserved grammatical motifs across species, providing a type of comparative syntax for DNA.

In fact, fundamental regulatory components like polyadenylation signals, TATA boxes, and CpG islands exhibit striking conservation across distant taxa, indicating that some “grammatical rules” are preserved because of their practical significance. Furthermore, species-specific innovations based on a shared syntactic basis are demonstrated by the use of alternative promoters in insects and plants and splicing mechanisms in vertebrates. Similar to how linguistic typologists compare sentence structures across languages, these conserved elements can act as anchors for comparative analyses, allowing non-coding regulatory grammars to align.

In addition to Zipf’s law, cross-species studies have incorporated other “linguistic” metrics such as Shannon entropy profiles and motif redundancy coefficients to assess the stability of these patterns across taxa. For example, comparative studies of mammalian, avian, and bacterial genomes show that although Zipf’s law metrics vary with genome size, the normalized Shannon entropy of nucleotide distributions remains within a narrow range in eukaryotes but is markedly lower in prokaryotes [38]. Similarly, the redundancy of functional motifs (e.g., TATA boxes, splice sites) shows a high degree of conservation within large clades, indicating the evolutionary stability of certain “syntactic” building blocks [39]. Moreover, the analysis of k-mer spectra in archaea, bacteria and eukaryotes reveals both universal patterns and taxon-specific distribution modalities, which allows for a formalized comparison of “genomic languages” [40].

According to recent comparative genomics research, organisms with more complex morphologies or cognitive processes are likely to have more complex genomic grammars. For example, mammalian genomes are distinguished by deeply nested regulatory architectures and long-range dependencies, while bacterial genomes frequently rely on linear, low-redundancy codon structures. This discrepancy might be an adaptive benefit of hierarchical and modular regulation in facilitating complex developmental programs and phenotypic plasticity. An interesting area for further study is whether cognitive characteristics across taxa are correlated with the complexity of DNA grammar.

## Experimental replication analysis

**Fig. 1:**
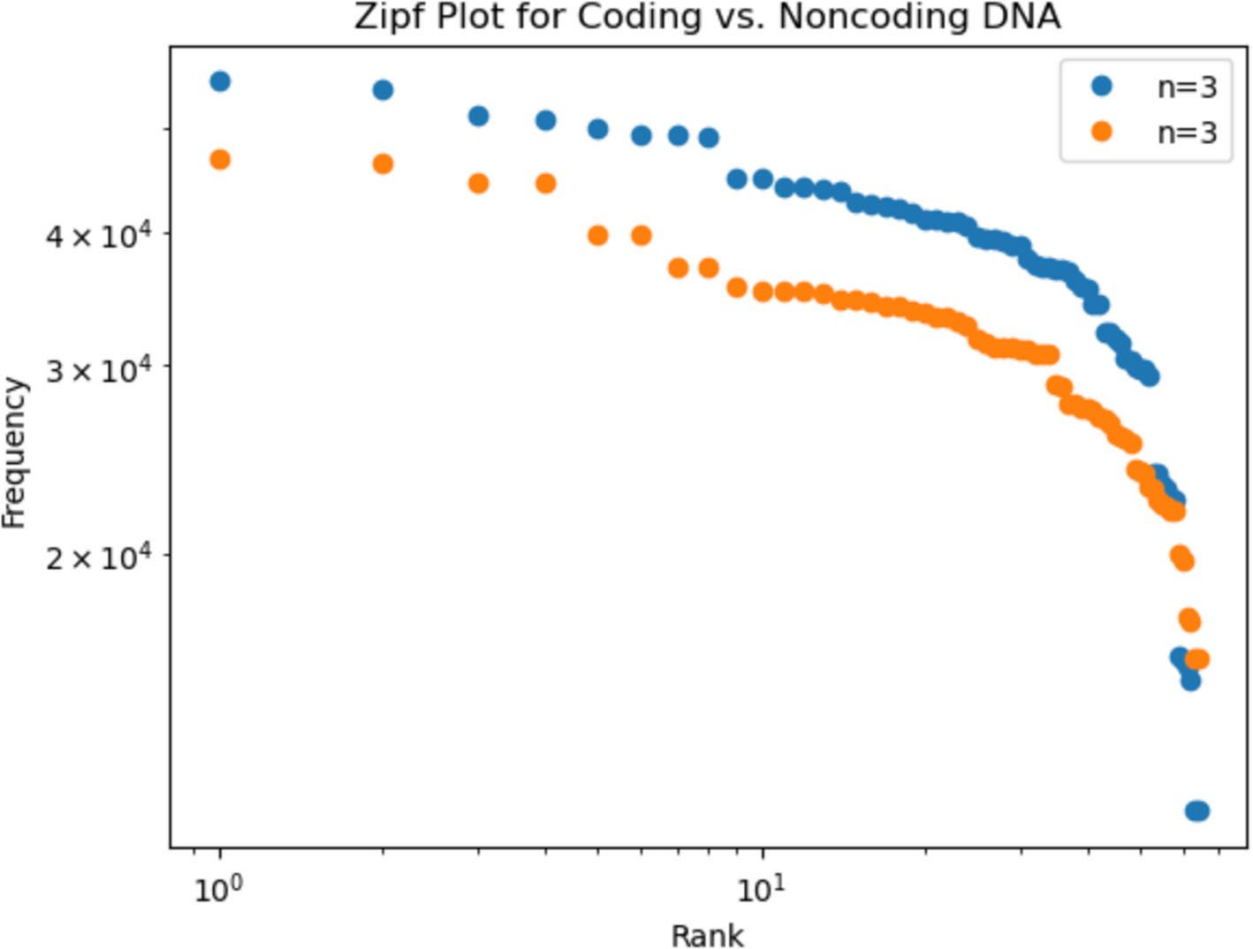
The replication analysis for Yarrowia lipolytica: non-coding DNA exhibits a power-law trend in Zipf analysis, whereas coding DNA deviates from a power-law behavior

To further validate the statistical models, an independent replication analysis was performed on Yarrowia lipolytica chromosome NC_090774. The sequence (4,198,534 bp) was downloaded in FASTA and GenBank formats, revealing 5298 annotated features. The coding regions summed to 2,292,132 bp, while the non-coding regions accounted for 1,906,542 bp. Zipf analysis (using 3-mers) on these separate regions demonstrated that non-coding DNA exhibits a clear power-law trend, with points aligning along a straight line in a log-log plot, in contrast to coding DNA which deviates from this behavior. Figure 1 displays the replication analysis results.

The hypothesis that non-coding DNA has linguistic-like statistical characteristics, consistent with a power-law distribution and high redundancy, is supported by this empirical finding. The existence of an underlying combinatorial grammar is suggested by Zipfian structure, which is represented by the alignment of non-coding 3-mer frequencies along a linear trend in the log-log space. The restricted and functionally optimized nature of protein-coding sequences, on the other hand, which depend on a fixed triplet codon system with little redundancy and a clearly defined mapping to amino acids, may be reflected in the deviation seen in coding regions.

These results are consistent with earlier research on the genomes of humans and mice and suggest that Zipfian behavior in non-coding DNA is not species-specific but rather could be an organizational principle in general. The idea that DNA’s “grammar” is an evolutionarily persistent trait is further supported by the successful replication on the unicellular eukaryote Yarrowia lipolytica, which indicates that such statistical features are conserved even in relatively simple organisms.

This methodology could be extended to other model organisms, such as prokaryotic species, Arabidopsis thaliana, or Drosophila melanogaster, in future replication efforts to investigate whether the degree of Zipfian regularity is correlated with organismal phenotype, regulatory density, or genomic complexity.

These results have biological implications that go beyond statistical regularity. An underlying regulatory architecture influenced by evolutionary forces that favor combinatorial reuse and mutational robustness is suggested by the presence of a strong Zipfian signal in non-coding DNA. Finding the k-mers that dominate the Zipfian distribution may help identify important motifs related to epigenetic control, enhancer-promoter dynamics, or transcriptional regulation.

Future research should incorporate statistical validation methods like bootstrapping, null model comparison, and power-law goodness-of-fit tests to increase the robustness of such analyses. Reproducibility across species and studies will be further enhanced by disclosing effect sizes, confidence intervals, and computational parameters like window size and k-mer step length. By following these procedures, it will be possible to make sure that the linguistic patterns found in DNA are authentic biological organization rather than methodological artifacts.

Together, these replication findings highlight DNA linguistics’ potential as a tool for comparison and reproducibility, opening the door for the difficulties covered in the next section.

## Challenges and future directions

Future research should also focus on (1) cross-species modeling using large-scale pretraining, as exemplified by models like the Nucleotide Transformer [7]; (2) hybrid symbolic-deep approaches for regulatory motif grammar induction [6]; and (3) interpretability frameworks that coalesce sequence statistics (e.g., entropy, Zipf) with model attention patterns to provide biologically meaningful explanations. There are still a number of obstacles in the way of completely understanding the genetic language, despite tremendous progress. To fully capture the intricacy of non-coding regulatory grammar, computational models need to be improved. Although methods like Transformer-based self-attention have made progress, more research is necessary to see whether they are applicable in a variety of genetic situations. Furthermore, thorough experimental validation is necessary when relating statistical characteristics (such as Zipfian distributions and long-range correlations) to particular biological activities. To improve model interpretability and biological relevance, future studies should investigate combining multi-omics data, doing cross-species comparisons, and creating common standards.

It may be possible to identify which linguistic traits are species-specific and which are universal by conducting comparative analysis across a variety of species, including prokaryotes, plants (Arabidopsis thaliana), insects (Drosophila melanogaster), and mammals. The expanded cross-species analysis presented in Section 9 already demonstrates that metrics beyond Zipf’s law—such as normalized entropy and motif redundancy—can capture conserved and divergent patterns across mammalian, avian, and bacterial genomes. Evolutionary gradients in the complexity of genomic grammar may be revealed by such analysis. The consistency of Zipfian patterns, entropy measures, and long-range correlations across various biological contexts would also be validated by replication studies across different chromosomes and organisms, taking into account variables like genome size, GC content, and repeat density.

Finally, results visualization from transformer-based models (e.g., DNABERT) can offer intuitive insight into genomic organization through the use of Zipf profiles, entropy landscapes, motif redundancy heatmaps, and attention maps. These visual aids improve interpretability and could reveal hidden regulatory structures that are not visible through black-box or purely statistical modeling techniques.

Furthermore, to guarantee the accuracy and repeatability of results, it is crucial to strengthen the methodological underpinnings of DNA linguistics. Future research should explicitly outline the metrics and algorithms used for statistical analysis, including long-range correlation detection, Shannon entropy estimation, and Zipf law fitting. The method used to evaluate statistical robustness, such as bootstrapping, null model comparisons, or the use of confidence intervals, should also be explained. Further improving transparency and facilitating cross-laboratory reproducibility can be achieved by explicitly reporting software environments and tools, such as Transformer frameworks like Hugging Face for DNALMs, R packages like zipfR and entropy, or Python libraries like Biopython and SciPy.

It is crucial to recognize the conceptual limitations of the linguistic metaphor, even though it has offered a strong framework for modeling genomic information. DNA lacks intentionality, semantic nuance, and cognitive context, in contrast to natural languages. Instead of originating from a communicative intent, the “meaning” of genomic motifs is the result of biochemical interactions and evolutionary optimization. Therefore, it is important to use linguistic models carefully so that biological specificity is not obscured by metaphorical parallels. The empirical requirements of molecular biology and the heuristic advantages of linguistic analogies must be balanced in future multidisciplinary endeavors.

### Conceptual limitations of the DNA–language analogy

Although linguistic frameworks offer a strong and user-friendly method for modeling genomic sequences, they are conceptually limited by nature. In contrast to natural languages, DNA lacks cognitive context, semantic subtleties, and communicative intent. Rather than syntactic intention, natural selection-shaped biochemical interactions give genetic motifs their “meaning.”

Overuse of linguistic metaphors runs the risk of simplifying or misrepresenting biological processes. For example, entropy patterns and grammatical rules reveal structure, but they do not always explain biological function or causality. In order to prevent metaphorical reasoning from overshadowing biological specificity, future research should strike a balance between the heuristic value of linguistic analogies and empirical validation from molecular biology.

## Cognitive and philosophical implications

There are interesting philosophical issues when considering DNA as a language. Does the “universal grammar” that governs the genome resemble the theory put forth by Noam Chomsky [10] in human linguistics? This idea is speculative, but it is somewhat supported by the recursive nature of genomic elements and the structural invariance seen across species. In addition, the the way that DNA encodes, transmits, and interprets information is similar to semiotic theories, which hold that context provides meaning to signs [19]. The similarities between the linguistic and genetic systems from a cognitive perspective include memory, hierarchy, and ambiguity. These links imply that there may be deeper evolutionary informational principles shared by the biological encoding of life and the human ability to speak.

On the other hand, knowledge from genomics may also be useful for linguistic theory. New approaches to modeling syntax or discourse may be sparked by the idea of modular regulatory motifs, which are recurring but context-dependent. Linguistic components like discourse markers or idiomatic expressions may operate through interaction networks influenced by higher-order structures, much like transcription factors bind motifs with context-specific outcomes. This interdisciplinary exchange may lead to new hybrid models in psycholinguistics and computational linguistics.

## Conclusion

A revolutionary paradigm for comprehending genome control and evolution is provided by DNA linguistics. Through the use of concepts from deep learning, statistical analysis, and formal language theory, scientists are starting to unravel the secret grammar underlying non-coding DNA. Furthermore, the co-evolution of language and genetic inheritance is empirically supported by genetic-linguistic relationships in human groups. The multidisciplinary combination of statistical physics, genetics, and linguistics shows great potential for expanding our knowledge of the language of life, even though there are still difficulties in improving computer models and confirming their biological roles.

Although the models and theories currently in use have greatly increased our understanding, they also bring up significant unanswered questions. For example, does every genome have its own distinct set of grammatical rules, or is there a universal genomic syntax shared by all species? These kinds of investigations push us to consider the functional significance of linguistic structures ingrained in the genome rather than just statistical regularities.

Furthermore, incorporating knowledge from multi-omics data—such as transcriptomics and epigenomics—may help future studies enhance DNA linguistic models with biological context. Finding long-range dependencies and regulatory patterns that might otherwise go undetected is made possible by the growing use of deep neural architectures, particularly transformer-based models. Recent additions to comparative analyses, including Shannon entropy profiles and motif redundancy coefficients across diverse taxa, further demonstrate that linguistic features can be quantitatively assessed and compared across species, strengthening the empirical foundation of the field.

Crucially, philosophical issues are also brought up in this field. By considering DNA as a language, we are redefining what it means to “read” and “interpret” life itself, in addition to analyzing biological data. We are prompted to reconsider the distinctions between meaning and information, code and communication, and biology and cognition by this linguistic metaphor.

According to this perspective, DNA linguistics is a bridge across disciplines that provides fresh perspectives on issues related to evolution, regulation, and the nature of life. It is more than just a computational framework.

## Data Availability

Sequence data used in this study were obtained from the NCBI Nucleotide database (accession number: NC_090774.1) and are publicly available at https://www.ncbi.nlm.nih.gov/nuccore/NC_090774.1. All processed data and analysis scripts used to generate Fig. 1 and Table 2 are available in the GitHub repository at https://github.com/puper26/Genomics-and-Informatics.

## Code availability

The code used for data processing and figure generation is available on GitHub at https://github.com/puper26/Genomics-and-Informatics/tree.
